# Does an open level 2 medical high-dependency unit improve outcomes for critically ill patients? Using the APACHE II scoring system in a district general hospital in the UK

**DOI:** 10.1186/cc9928

**Published:** 2011-03-11

**Authors:** V Hurley, D Pandit

**Affiliations:** 1 Russells Hall Hospital, Birmingham, UK

## Introduction

Improving care of acutely ill medical patients led to formation of a six-bed level 2 medical high-dependency unit (MHDU). The aim of this study was to look at outcomes of medical patients admitted to an open level 2 MHDU.

## Methods

One hundred and nine patients were consecutively admitted to the MHDU in a prospective observational study. APACHE II derived mortality scores averaged for two groups of patients - those who survived the admission and those who did not - and were assessed using a chi-squared test.

## Results

A total 48.6% of patients were male, mean age 59.3 years (range 0 to 98 years). Average total length of stay in hospital was 16.55 days with average 4.29 days in the MHDU (range 0 to 18 days). In total, 34.9% admissions were respiratory in origin, 22% sepsis, 10% GI, 7.4% poisonings, 5.5% other, 4.6% renal, 3.6% cardiac, 2.8% neurological and <1% unclassified. A total 29.3% of patients were admitted directly from A&E, 37.6% from the emergency admissions unit and 33% from the wards (27% of these from ITU). Two per cent of patients required ITU admission after the MHDU. Twenty-two patients out of 109 died during this admission, 13 of them while admitted to the MHDU. Deaths were classified according to diagnosis on admission to the MHDU, with 45% with GI disease dying, 29% with sepsis, 22% endocrine and 21% respiratory. These patients were deemed not suitable for escalation to level 3 care. Of 109 patients, full APACHE II data were available for 87. Of this subcohort, 16 patients died and 71 survived. Expected values were calculated and predicted that 26 should have died and 61 survived (*P *< 0.05) from the APACHE II data.

## Conclusions

The cost of NHS care is becoming increasingly important in the UK and anecdotal evidence suggests a high proportion of patients managed in level 3 care could more appropriately be managed with a lower level of care ideally in an HDU setting, while decisions can be made whether the physiological status of the patient justifies escalation of care. This observational study raises questions about appropriateness of admission to MHDU and has led to improvement of gatekeeping to the unit. This study also demonstrates increasing involvement of critical care in managing end-of-life challenges. We have used this study to demonstrate to our colleagues what critical care can and cannot offer. Future studies to characterise performance of our unit will use the SAPs and risk profile management method.
